# Experiencing Novelty in Adolescence and the Influence of Prior Novelty-Related Experiences on Adult Behavioral Outcomes in Wistar Han Rats

**DOI:** 10.3390/ani15243552

**Published:** 2025-12-10

**Authors:** Maja Srbovan, Milica Potrebić Stefanović, Olga Dubljević, Željko Pavković

**Affiliations:** Laboratory of Molecular Neurobiology and Behavior, Department of Neurobiology, Institute for Biological Research “Siniša Stanković”—National Institute of Republic of Serbia, University of Belgrade, 11060 Belgrade, Serbia; maja.srbovan@ibiss.bg.ac.rs (M.S.); milica.potrebic@ibiss.bg.ac.rs (M.P.S.); olga.dubljevic@ibiss.bg.ac.rs (O.D.)

**Keywords:** adolescence, prior novelty-related experience, test–retest, exploratory behavior, risk-taking, anxiety-like behavior

## Abstract

Acquiring new experiences, especially in adolescence, shapes behavioral responses to life’s challenges. The absence of novelty-related experiences in laboratory rats raises concerns about their behavioral development and the reproducibility of research. This study examined changes in behavioral responses to novelty from early and late adolescence to adulthood, as well as the long-term effects of prior novelty exposure. To assess maturational differences, seven early adolescent, nine late adolescent, and seven adult male Wistar Han rats were tested once in a behavioral battery consisting of the novel arena, novel object exploration, light/dark box, and elevated plus maze. To evaluate the influence of prior experience, early and late adolescent groups were retested once more in adulthood. Late adolescents showed consistent patterns across spatial tasks, displaying increased anxiety-like behavior and reduced exploratory and risk-assessment activity. Early adolescents exhibited variable behaviors, resembling adult or late adolescent profiles depending on the task. Novelty exposure during adolescence produced subtle long-term effects, including reduced discomfort in mildly aversive environments, while late adolescent experience increased thigmotaxis in adulthood. Overall, adolescent responses to novelty were influenced more by spatial than nonspatial environmental features. These findings highlight stage-specific sensitivity to novelty, the influence of adolescent experience, and the potential reuse of experienced animals.

## Simple Summary

Laboratory animals are raised in controlled environments deprived of any novelty-related experiences, which raises questions about how the lack of such experiences, particularly during adolescence, affects typical animal behavioral development. Utilizing the most commonly used ethological tests designed to evaluate exploratory, risk-taking, and anxiety-like behavior in rodents, we assessed the behavioral profile during maturation of male rats from early and late adolescence to adulthood, as well as the influence of prior novelty-related experience acquired during adolescence on behavioral outcomes in adulthood. Our results revealed a pronounced apprehensive response to novelty exposure in late adolescence and a more diffuse behavioral outcome in early adolescence, featuring elements of either late adolescent or adult responses, depending on the environmental setting to which they were exposed. Adolescent novelty exposure led to subtle behavioral changes in adulthood in environments with lower anxiogenic potential. Generally, spatial rather than nonspatial environmental factors influenced adolescents’ responses to novelty. This study highlights the significance of experiencing spatial novelty during adolescence as well as the potential benefits of such an experience, and suggests the possibility of reusing animals with prior experience in subsequent experiments.

## 1. Introduction

Life in natural habitats implies exposure to various new environmental stimuli, which enable the acquisition of necessary experiences that shape future behavioral reactions to the inconsistencies of everyday life. However, laboratory animals are raised in environmentally controlled cages, a setting characterized by confinement, monotony, and lack of challenge and accompanied by boredom, a condition linked to behavioral abnormalities [1,2]. Consequently, a particular question emerges of whether the lack of novelty-related experiences in animals housed in such conditions influences specific developmental changes that may contribute to the poor reproducibility and translational value of the research results using rodents [3,4].

Novelty is defined as the process of experiencing something different from something usual or familiar [5]. In response to novel stimuli, animals exhibit two evolutionarily conserved yet distinct behavioral patterns: neophilia (approach) and neophobia (avoidance) [6]. These behaviors are shaped by the nature of the novelty—whether the stimulus is entirely unfamiliar (absolute novelty), shares characteristics with previous experiences (contextual or common novelty), or has already been memorized after repeated exposures (familiar stimulus) [2,7]. Rodents usually show a preference for novelty as they tend to explore novel places and objects more than familiar ones [8,9], but also an emotional reaction, such as anxiety, if the stimulus is perceived as unpleasant or aversive [10,11]. This approach/avoidance paradigm is utilized in many well-validated ethological tests used to assess behavioral/emotional responses to the spatial (novel arena—NA, light/dark box—LDB, elevated plus maze tests—EPM) or nonspatial (novel object exposure test—NOE) aspects of environmental novelty. In NA, the basic test of motor activity in rodents, animals tend to explore open space in search of an exit, usually avoiding the center area and staying in close proximity to the walls (thigmotaxis), which is considered a measure of anxiety-like behavior [12]. By introducing an object in such a familiar environment (NOE), scientists can assess animals’ conflict of exploring or avoiding nonspatial stimuli [13]. However, the LDB and EPM are the most widely used tests for assessing risk-taking and anxiety-like behavior in rodents [14,15]. They are based on the natural tendency of nocturnal animals to seek shelter in dark areas and avoid brightly lit compartments in the LDB, as well as to avoid the highly aversive open and elevated arms of the EPM, which represent areas of high risk of predator attack.

Responses to novel stimuli are also largely influenced by the stage of development. Adolescence is widely recognized as a critical period characterized by increased risk-taking and enhanced exploratory behavior. Accordingly, some studies suggest that adolescent animals exhibit heightened locomotor responses to novelty, an increased preference for novel stimuli, and more pronounced approach and exploratory behaviors relative to adults [16]. In humans, adolescence is similarly marked by emotional instability, increased impulsivity, and a heightened tendency toward risk-taking, factors that have been associated with an elevated vulnerability to various neuropsychiatric disorders, including mood disorders and substance abuse [17,18,19]. Given the behavioral changes and key social transitions of adolescence, this developmental stage is hypothesized to involve heightened stress sensitivity and increased anxiety relative to childhood and adulthood [18]. These behavioral traits are generally understood to arise from the ongoing maturation of neural networks involved in emotional regulation and inhibitory control [20,21,22]. Overall, while these maturational changes are viewed as ontogenetic adaptations that enhance the ability to navigate the transition from childhood to adulthood and the pursuit of independence, they simultaneously increase adolescents’ susceptibility to diverse environmental influences [18,23].

Previous research on rodents has demonstrated that adolescent animals respond differently to novelty compared to adults, showing variations in fear-, anxiety-, and depression-related behaviors as well as stress reactivity (see reviews, [22,24]). Specifically, adolescent rats have been reported to exhibit anxiety-like behavior that is sometimes lower, higher, or comparable to that observed in adult rats across different behavioral tests [24,25,26]. Moreover, it has been proposed that prior exposure to the elevated plus maze alters the animal’s emotional state in a way that leads to the development of a conditioned phobic response rather than a purely innate anxiety reaction [27,28]. Although a 4-week “washout” period is considered sufficient for stable elevated plus maze responses upon retest in adult Wistar rats [29], it remains unclear whether prior experience in novel environments during specific adolescent stages influences long-term behavioral outcomes.

To our knowledge, no efforts have been made to elaborate on this issue. Studies were mainly focused on the effects of adverse experiences, such as physical or social restraint, wherein outcomes of adolescent adversity differ depending on the maturational stage in which the adversity occurs [30]. However, a novel environment is a mild stressor, as it induces only moderately elevated corticosterone levels in rats [31]. Since stress levels in a novel environment are lower than those caused by induced stress (such as chronic restraint) [32], a different behavioral outcome is also plausible. Moreover, it is shown that exposure to acute mild stressors can even stimulate increased brain plasticity and enhance cognitive performance [33].

Considering all the aforementioned, the present study aimed to assess behavioral/emotional response to novelty from early and late adolescence to adulthood, with the specific goal to examine whether novelty-related experience acquired at a particular adolescent stage influences later behavioral/emotional reactivity. We hypothesized that behavioral responses to novelty may vary across developmental stages and that prior exposure to novelty during adolescence can lead to subtle long-term behavioral alterations. Results from this study reveal stage-specific behavioral response to novelty, which specific aspects of emotional/behavioral response to novelty are influenced by prior adolescent experience, and whether animals with prior novelty-related experience can be adequately used in later experiments.

## 2. Materials and Methods

### 2.1. Animals

All experiments were carried out using 27 male Wistar Han rats selected from 9 different litters. Animals were bred and raised in the accredited Animal Facility of the Institute of Biological Research “Siniša Stanković”—National Institute of Republic of Serbia, University of Belgrade, Serbia, in controlled housing conditions (a room temperature 22 ± 1 °C, relative humidity 50 ± 5%, 12 h light/dark cycle with lights on at 7:00 a.m., standard cages of enclosure size 800 cm^2^ (European standard Type 3H, ZOONLAB, Castrop-Rauxel, Germany), made from transparent plastic). Autoclaved wood shavings as bedding material (PREMIUMSPAN^®^, HVT Hobelspanverarbeitung GmbH, Dittersdorf/Thüringen, Germany) were provided in an amount sufficient to cover the floor. The home cages were placed side by side so rats could smell, see, and hear each other. No cages containing female rats were placed near the cages with experimental male rats. Tap water and standard pelleted food (Gebi Doo, Cantavir, Serbia) were available ad libitum. Environmental enrichment was not provided in order to avoid potential confounding effects on study design. Animals were monitored daily and showed no signs of illness or distress. No humane endpoints were established for this study.

All animal procedures complied with the policies on animal welfare and were approved by the Ethical Committee of the Institute and the National Ethics Research Committee (323-07-03378/2023-05).

### 2.2. Experimental Procedure

At postnatal day (PND) 21, animals were separated from their mothers, and 3 male rats from the same litter were marked for recognition and placed together in the home cage for further housing (one cage per litter, 3 siblings per cage). Marking was performed using a 1% aqueous solution of picric acid, which leaves a long-lasting yellow mark on white fur. This safe and widely used method avoids more invasive or stressful procedures, such as ear-notch marking. Behavioral response to novelty during maturation of Wistar Han rats was assessed by exposing randomly selected animals from the same cage to a battery of behavioral testing at the age PND 35 ± 1, PND 49 ± 1, or PND 155 ± 1. These postnatal days are defined in earlier studies as maturational stages in rats that typically correspond to early (i.e, early to mid) adolescence (EA), late adolescence (LA), and adulthood (A), respectively [34]. In this manner, three experimental age groups were established and counterbalanced by litter, ensuring that each litter contributed one male to each group (i.e., one animal per group per breeding pair), thereby preventing potential maternal effects. The group sizes were determined based on our previous experience with the behavioral tests used in this study [35,36,37], as well as on comparable methodological approaches reported in other studies [38,39,40,41,42]. This approach aimed to minimize the number of animals used while still obtaining statistically meaningful results, in accordance with the principle of reducing animal use. The battery of non-invasive behavioral tests was comprised of the novel rectangular arena (NA, 20 min), novel object exploration in the familiar arena (NOE, 10 min), light/dark box (LDB, 10 min), and elevated plus maze (EPM, 10 min) tests to assess ontogeny of anxiety-like behavior, as well as risk-assessment, exploratory, and stereotypy-like behavior. All age groups of animals underwent tests in the above order (NA-NOE, LDB, EPM), based on established methodologies reported in the related literature [25,38,39,40] and to minimize the risk that exposure to prior more stressful tasks would influence baseline behavior in subsequent tests [43,44]. Prior to each behavioral test, animals were acclimated to the experimental room for 30 min. Tests were conducted over 3 consecutive days (one test per day) between 9 a.m. and 2 p.m. Following this period, the tested animals were returned to their home cages in accordance with the previously defined housing conditions. The equipment was cleaned to eliminate any scent traces from previously used animals, prior to the following testing. The influence of novelty-related experience acquired in adolescence on behavior in adulthood was evaluated by retesting animals that underwent the test procedure in early and late adolescence later in adulthood (PND 155 ± 1), and comparing them to inexperienced adults. All animal handling was performed by one experimenter. Blinding was implemented during video recording analysis, as well as during statistical analysis of the obtained data. All experiments were performed in the Laboratory for Molecular Neurobiology and Behavior, Department of Neurobiology, Institute for Biological Research “Siniša Stanković”—National Institute of the Republic of Serbia, University of Belgrade, Serbia.

### 2.3. Exclusion and Termination Criteria

During the implementation of this experimental study, we followed the rules of the ARRIVE guidelines regarding exclusion and termination criteria [45]. All experimental subjects were alive and in good health throughout the duration of the study. In addition, special attention was given to minimize potential distress during animal handling. However, due to technical difficulties with camera freezing, 2 animals from the early adolescent group and 2 from the adult group had to be excluded from further analysis at the end of behavioral testing. Thus, the final number of animals per group was **7** for early adolescence, **9** for late adolescence, and **7** for adulthood.

### 2.4. Novel Rectangular Arena and Novel Object Exploration Test Procedures

Experimental procedure and equipment for measuring rat behavior in the NA and NOE test were described in detail in a previously published study of our group [35].

Motor activity of each animal was recorded by automated equipment comprised of Opto-Varimex cages (Columbus Instruments, Columbus, OH, USA) that were linked on-line to an IBM-PC compatible computer. Animals were placed in the center of the arena (30 min after the habituation of animals in home cages to the room conditions) and allowed to freely explore the novel environment for 20 min. Data were analyzed using Auto-Track software, version 4.51 (Columbus Instruments). Locomotor activity was defined as a trespass of consecutive infrared beams (total distance traveled), stereotypy-like movements as the number of repeated breaks of the same beam (includes body rocking, head movement, repetitive paw movements such as grooming, shaking, and tapping), and vertical activity as the number of infrared beams that were broken by the rearing of the animal. Auto-Track interface has the ability to detect movements in 16 (4 × 4) equal fictional squares of the arena and to record time spent in each square. Rats usually avoid central (anxiogenic) areas (thigmotaxis). After exploration and familiarization with the testing arena, rats were briefly returned to their home cage, and during this time, an object (plastic bottle 5 × 5 cm in the base and 10 cm in height, filled with sand to increase the weight of the object) was placed into the center of the arena. Thereafter, the animal was gently placed in the corner of the arena to freely explore the object for an additional 10 min. Behavior during the object exploration was recorded by a camera, providing material for subsequent analysis by a researcher not familiar with the experimental design. Parameters that were included in the analysis are latency to novel object approach, number of approaches, and time spent in the novel object exploration. An exploration was defined as a situation when the animal approaches the object at 2 cm or less (head forward).

### 2.5. Light/Dark Box Test Procedure

Experimental procedure and equipment for measuring rat behavior in the LDB were described in detail in a previously published study of our group [36].

After 30 min of habituation to the room conditions (in home cages), one animal was placed in the middle of the light compartment facing opposite the side in which the door was located and left to freely explore the maze for the following 10 min. Activity of each animal was recorded by a camera, allowing subsequent analysis of the recorded material by a researcher not familiar with the experimental design. The parameters included in the analysis were latency to enter the dark compartment, number and time spent in stretch-attended postures (i.e., posture where the body is extending into the illuminated part, however, not all four paws are located in the illuminated section), number of entries into the light compartment, and time spent in the light and dark compartments, respectively (measured for the time when all four paws of the animal are present in the specific compartment/section).

### 2.6. Elevated Plus Maze Test Procedure

Experimental procedure and equipment for measuring rat behavior in the EPM were described in detail in a previously published study of our group [37]. For the purpose of this study, we made an additional adjustment by dividing the open arms into three equal segments and analyzing the time spent in the most distal third versus the proximal two-thirds. Because the distal third of the open arms is reported to be more aversive than the proximal segments, this approach may provide more detailed information, as explained in a related study [35].

Briefly, after 30 min of habituation to the room conditions (in home cages), one animal was placed in the center and left to freely explore the maze for the next 10 min. Activity of each animal was recorded by a camera, allowing subsequent analysis of the recorded material by a researcher not familiar with the experimental design. The parameters included in the analysis were latency to enter closed area, number and time spent in stretch-attended postures, time spent in the center, number of entries in the closed compartment, number of entries in the open area, total number of entries, time spent in the first two-thirds of the open arms and time spent in the most distal third of the open arm. Preference based on the number of entries in the open area was defined as a proportion of open arm entries to the total number of entries. Open arms were divided into three equal sections to allow for a better understanding of how animals experience zones with different anxiogenic potential, bearing in mind that higher anxiety was reported on distal compared to proximal positions in the open arms [46]. With these reports in mind, it is reasonable to assume that the proportion of open arm entries to the total number of entries, without insight into the retention in certain parts of the open arms, is, in essence, incomplete information [35].

### 2.7. Statistical Analysis

The obtained data were analyzed using Statistica 12 software (StatSoft Inc., Tulsa, OK, USA), and the following results are presented as means ± SD, with individual data plots along the column bars. The normality of data sets was estimated by the Shapiro–Wilk test. The accepted level of significance was *p* ≤ 0.05 for all tests. The statistical results, along with the tests used, and the exact *p*-values are summarized in Supplementary Tables S1 and S2.

The data obtained for the purpose of observing the differences in exploratory and anxiety-like response to novelty during maturation and effects of previous exposure to novelty that possessed a normal distribution were analyzed by one-way ANOVA, followed by a post hoc unequal N HSD test. Data that did not have a normal distribution were analyzed via the Kruskal–Wallis H test, followed by the Mann–Whitney U test for pairwise comparisons. To control the overall false discovery rate, a global Benjamini–Hochberg (BH) correction was applied to all test parameters included in the analysis. To confirm the preference for the number of entries in open arms of EPM, each group was compared to the chance levels (0.5) using one-sample *t*-test.

## 3. Results

### 3.1. Behavioral Response to Novelty Exposure During Maturation

#### 3.1.1. Behavioral Profile in Novel Rectangular Arena Test During Maturation

**Figure 1:**
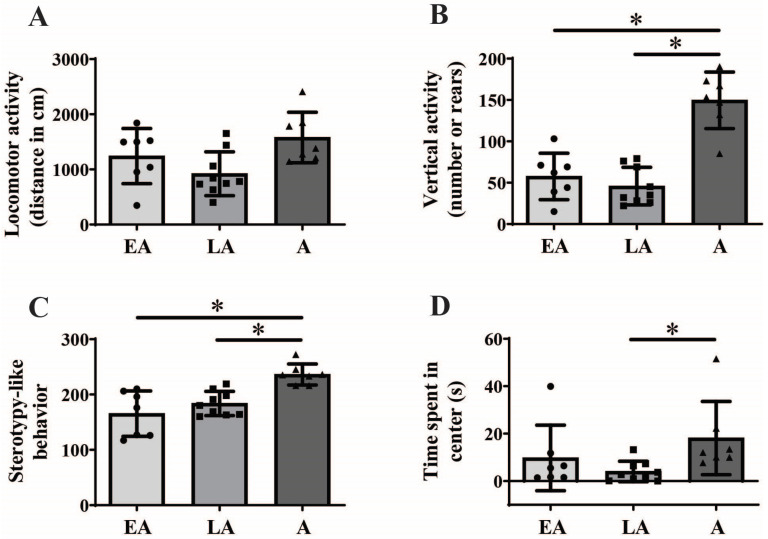
Behavioral response to novel rectangular arena exposure from early adolescence (EA) and late adolescence (LA) to adulthood (A). The data are represented as mean ± SD, with individual data plots along the column bars. Parameters analyzed were locomotor activity, assessed through total distance traveled (**A**), vertical activity, assessed through total number of rears (**B**), stereotypy-like activity (**C**), time spent in the center (**D**). Statistical significance: * if *p* ≤ 0.05. Statistical comparisons and exact *p*-values are provided in Supplementary Table S1.

One-way ANOVA analysis followed by BH correction showed maturational differences in vertical activity (Figure 1B; F_(2,20)_ = 30.226; *p* < 0.001, BH corrected *p* = 0.007) and stereotypy-like behavior (Figure 1C; F_(2,20)_ = 11.895; *p* < 0.001, BH corrected *p* = 0.007), but not in locomotor activity (Figure 1A; F_(2,20)_ = 4.257; *p* = 0.029, BH corrected *p* = 0.067). Kruskal–Wallis ANOVA followed by BH correction showed maturational differences regarding time spent in the center of the NA (Figure 1D, H_(2, N = 23)_ = 8.966, *p* = 0.011, BH corrected *p* = 0.035).

Post hoc analysis revealed that adult animals display higher vertical activity and stereotypy-like behavior compared to both early (Figure 1B, * *p* < 0.001 and Figure 1C, *p < 0.001) and late adolescent animals (Figure 1B, * *p* < 0.001 and Figure 1C, * *p* = 0.007), respectively. Furthermore, adult animals spent more time in the center of NA compared to late adolescent animals (Figure 1D, U test, * *p* = 0.004). There were no significant differences between early and late adolescent animals in all examined parameters.

#### 3.1.2. Behavioral Profile in the Novel Object Exploration Test During Maturation

**Figure 2:**
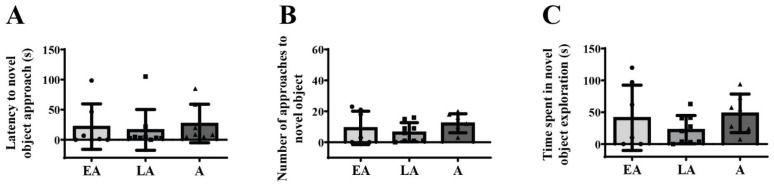
Behavioral response to the novel object exposure in a familiar arena from early adolescence (EA) and late adolescence (LA) to adulthood (A). The data are represented as mean ± SD, with individual data plots along the column bars. The following parameters were analyzed: latency to novel object approach (**A**), number of approaches to the novel object (**B**), and the amount of time spent in the novel object exploration (**C**). No statistically significant differences were observed. Statistical comparisons and exact *p*-values are provided in Supplementary Table S1.

No significant differences were observed in latency to NO approach (Figure 2A), the number of approaches to NO (Figure 2B), and time spent in NO exploration (Figure 2C) between all analyzed groups.

#### 3.1.3. Behavioral Profile in Light/Dark Box Test During Maturation

**Figure 3:**
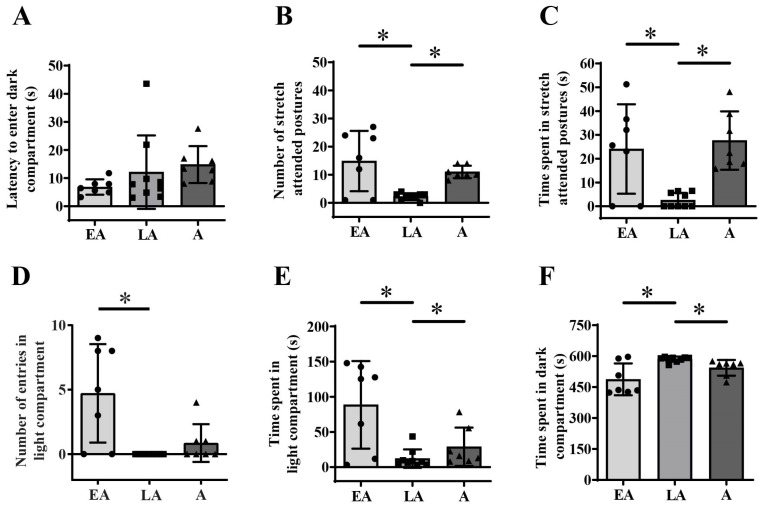
Behavioral response to light/dark box exposure from early adolescence (EA) and late adolescence (LA) to adulthood (A). The data are represented as mean ± SD, with individual data plots along the column bars. Parameters analyzed were latency to enter dark compartment (**A**), the number of stretch-attended postures (**B**), the amount of time spent in the stretch-attended postures (**C**), the number of entries in the light compartment (**D**), the amount of time spent in the light compartment (**E**), the amount of time spent in the dark compartment (**F**). Statistical significance: * if *p* ≤ 0.05. Statistical comparisons and exact *p*-values are provided in Supplementary Table S1.

Kruskal–Wallis ANOVA followed by BH correction showed maturational differences in the number of stretch-attended postures (Figure 3B, one-way ANOVA, F_(2,20)_ = 9.285; *p* = 0.001, BH corrected *p* = 0.007) but no in the latency to enter the dark compartment of LDB (Figure 3A, H_(2, N = 23)_ = 6.932, *p* = 0.031, BH corrected *p* = 0.068). Kruskal–Wallis ANOVA followed by BH correction also revealed maturational differences in the time spent in stretch-attended postures (Figure 3C, H_(2, N = 23)_ = 10.433; *p* = 0.005, BH corrected *p* = 0.022), number of entries into the light compartment (Figure 3D, H_(2, N = 23)_ = 9.906; *p* = 0.007, BH corrected *p* = 0.026), time spent in the light compartment (Figure 3E, H_(2, N = 23)_ = 9.892; *p* = 0.007, BH corrected *p* = 0.026) and time spent in the dark compartment (Figure 3F, H_(2, N = 23)_ = 8.224; *p* = 0.016, BH corrected *p* = 0.047) of LDB.

Post hoc unequal N (HSD) analysis showed a decrease in the number of stretch-attended postures from early to late adolescence (Figure 3B, * *p* = 0.003) and an increase from late adolescence to adulthood (Figure 3B, * *p* = 0.034). Decrease from early to late adolescence (Figure 3C, U test, * *p* = 0.005) and an increase from late adolescence to adulthood (Figure 3C, * *p* = 0.001) were also observed for time spent in stretch-attended postures. The number of entries into the light compartment differed only between early and late adolescent animals and was lower in late adolescence (Figure 3D, U test, * *p* = 0.017). Additionally, post hoc analysis showed a decrease in time spent in the light compartment from early to late adolescence (Figure 3E, U test, * *p* = 0.023) and an increase later in adulthood (Figure 3E, * *p* = 0.039), while for time spent in the dark compartment, it showed an increase in late adolescence (Figure 3F, U test * *p* = 0.039) and a decrease thereafter (Figure 3F, * *p* = 0.006).

#### 3.1.4. Behavioral Profile in the Elevated Plus Maze Test During Maturation

**Figure 4:**
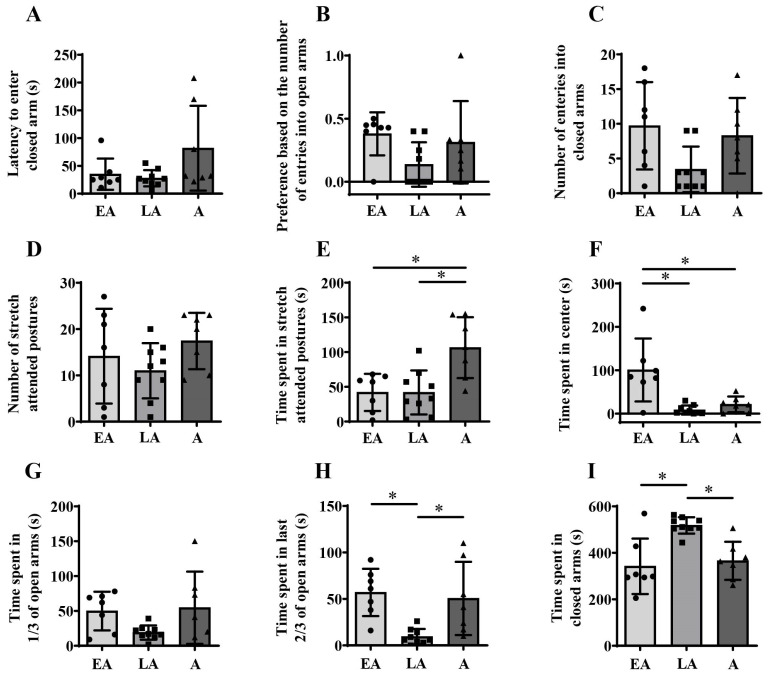
Behavioral response to elevated plus maze exposure from early adolescence (EA) and late adolescence (LA) to adulthood (A). The data are represented as mean ± SD, with individual data plots along the column bars. Parameters analyzed were latency to enter closed arm (**A**), preference based on the number of open arm entries (**B**), number of entries in closed arms (**C**), number of stretch-attended postures (**D**), time spent in stretch-attended postures (**E**), time spent in center (**F**), time spent in 1/3 of open arms (**G**), time spent in last 2/3 of the open arms (**H**), and time spent in the closed arms (**I**). Statistical significance: * if *p* ≤ 0.05. Statistical comparisons and exact *p*-values are provided in Supplementary Table S1.

Kruskal–Wallis ANOVA followed by BH correction showed no significant differences during maturation in latency to enter the closed arms of EPM (Figure 4A) and number of entries in closed arms (Figure 4C), and in the preference based on the number of entries in open arms (Figure 4B, H_(2, N = 23)_ = 7.129; *p* = 0.028, BH corrected *p* = 0.067). One-way ANOVA followed by BH correction showed no significant differences during maturation in the number of stretch-attended postures (Figure 4D), while significant changes were observed in the time spent in stretch-attended postures (Figure 4E, F_(2,20)_ = 8.502; *p* = 0.002, BH corrected *p* = 0.011) and time spent in the center (Figure 4F, F_(2,20)_ = 11.508; *p* = 0.001, BH corrected *p* = 0.007). For the parameter time spent in first third of open arms one-way ANOVA showed no significant changes during maturation in EPM (Figure 4G), but revealed significant maturational differences in time spent in the last two-thirds of open arms (Figure 4H, F_(2,20)_ = 7.967; *p* = 0.003, BH corrected *p* = 0.015) and time spent in the closed arms (Figure 4I, F_(2,20)_ = 11.029; *p* = 0.001, BH corrected *p* = 0.007).

Post hoc analyses showed a higher time spent in stretch-attended postures in adulthood compared to both early adolescence (Figure 4E, unequal N (HSD), * *p* = 0.006) and late adolescence (Figure 4E, * *p* = 0.006). In contrast, early adolescent animals spent more time in the center of EPM compared to both late adolescent (Figure 4F, unequal N (HSD), * *p* = 0.001) and adult animals (Figure 4F; * *p* = 0.008). There were no differences between age groups in time spent in the first third of the open area (Figure 4G), but there was a decrease in time spent the last two-thirds of the open area from early to late adolescence (Figure 4H; unequal N (HSD), * *p* = 0.008) followed by an increase thereafter (Figure 4H; * *p* = 0.021). In contrast, an increase in time spent in the closed area from early to late adolescence (Figure 4I; * *p* = 0.002) was followed by a decrease from late adolescence to adulthood (Figure 4I; * *p* = 0.007).

### 3.2. Influence of Prior Adolescent Novelty-Related Experience on Behavioral Outcomes in Adulthood

#### 3.2.1. Influence of Prior Adolescent Novelty-Related Experience on Later Behavioral Outcomes in the Novel Rectangular Arena Test

**Figure 5:**
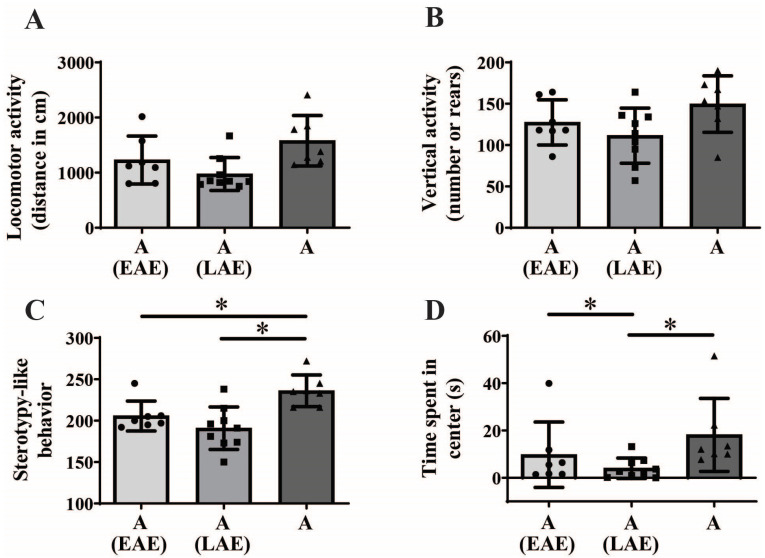
Influence of novelty-related **e**arly **a**dolescent **e**xperience (EAE) or **l**ate **a**dolescent **e**xperience (LAE) on adult (A) behavioral changes in the novel rectangular arena. The data are represented as mean ± SD, with individual data plots along the column bars. Parameters analyzed were locomotor activity, assessed through total distance traveled (**A**), vertical activity, assessed through total number of rears (**B**), stereotypy-like activity (**C**), and time spent in the center (**D**). Statistical significance is presented as * if *p* ≤ 0.05. Statistical comparisons and exact *p*-values are provided in Supplementary Table S2.

Kruskal–Wallis ANOVA followed by BH correction showed a significant influence of prior adolescent novelty-related experience time spent in center (Figure 5D, H_(2, N = 23)_ = 9.981; *p* = 0.001, BH corrected *p* = 0.007), but not on locomotor activity during adulthood in NA (Figure 5A, H_(2, N = 23)_ = 7.882; *p* = 0.019, BH corrected *p* = 0.052). While one-way ANOVA showed no influence of prior adolescent experience on vertical activity (Figure 5B), it showed a significant influence of prior adolescent novelty-related experience on stereotypy-like activity during adulthood (Figure 5C, F_(2,20)_ = 8.681; *p* = 0.002, BH corrected *p* = 0.011).

Post hoc analysis revealed that adults without previous adolescent experience exhibited higher stereotypy-like activity compared to both animals with prior early adolescent experience (Figure 5C, unequal HSD test, * *p* = 0.042) and late adolescent experience (Figure 5C, unequal HSD test, * *p* = 0.003). Adult animals with late adolescent novelty-related experience spent less time in the center compared to both adults with early adolescent experience (Figure 5D, U test, * *p* = 0.006) and adults without prior adolescent experience (Figure 5D, U test, * *p* = 0.020).

#### 3.2.2. Influence of Prior Adolescent Novelty-Related Experience on Later Behavioral Outcomes in the Novel Object Exploration Test

**Figure 6:**
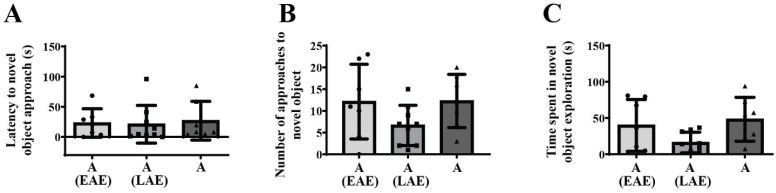
Influence of novelty-related **e**arly **a**dolescent **e**xperience (EAE) or **l**ate **a**dolescent **e**xperience (LAE) on adult (A) behavioral outcome in the novel object exploration test. The data are represented as mean ± SD, with individual data plots along the column bars. Parameters analyzed were latency to novel object approach (**A**), number of approaches to the novel object (**B**), and the amount of time spent in the novel object exploration (**C**). No statistically significant differences were detected. Statistical significance: Statistical comparisons and exact *p*-values are provided in Supplementary Table S2.

The statistical analysis did not reveal a significant influence of adolescent novelty-related experience on latency to approach the NO (Figure 6A), the number of approaches to NO (Figure 6B), and time spent in NO exploration (Figure 6C).

#### 3.2.3. Influence of Prior Adolescent Novelty-Related Experience on Later Behavioral Outcomes in the Light/Dark Box Test

**Figure 7:**
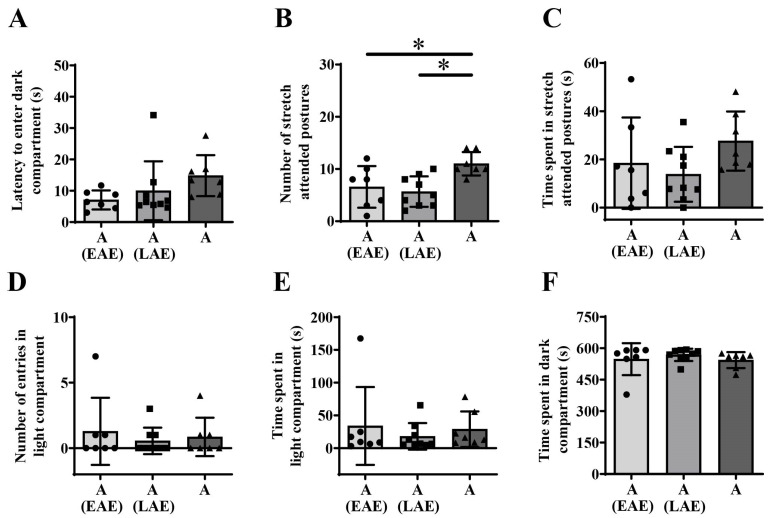
Influence of novelty-related **e**arly **a**dolescent **e**xperience (EAE) or **l**ate **a**dolescent **e**xperience (LAE) on adult (A) behavioral outcome in the light/dark box test. The data are represented as mean ± SD, with individual data plots along the column bars. Parameters analyzed were latency to enter dark compartment (**A**), number of stretch-attended postures (**B**), the amount of time spent in the stretch-attended postures (**C**), number of entries in the light compartment (**D**), the amount of time spent in the light compartment (**E**), and the amount of time spent in the dark compartment (**F**). Statistical significance is presented as * if *p* ≤ 0.05. Statistical comparisons and exact *p*-values are provided in Supplementary Table S2.

Kruskal—Wallis ANOVA followed by BH correction showed no significant influence of prior novelty-related adolescent experience on latency to enter the dark compartment (Figure 7A, H_(2, N = 23)_ = 7.623; *p* = 0.022, BH corrected *p* = 0.052), while one-way ANOVA revealed a significant influence of adolescent experience on the number of stretch-attended postures (Figure 7B, F_(2,20)_ = 6.295; *p* = 0.008, BH corrected *p* = 0.027). Kruskal–Wallis ANOVA followed by BH correction revealed no significant influence of prior adolescent novelty-related experience on time spent in stretch-attended postures (Figure 7C), the number of entries into the light compartment (Figure 7D), time spent in the light compartment (Figure 7E), and time spent in the dark compartment of LDB (Figure 7F).

Post hoc analysis showed that inexperienced animals display higher number of stretch-attended postures compared to animals exposed to novel environments in early adolescence (Figure 7B, unequal N(HSD), * *p* = 0.038) or late adolescence (Figure 7B, * *p* = 0.012).

#### 3.2.4. Influence of Prior Adolescent Novelty-Related Experience on Later Behavioral Outcome in the Elevated Plus Maze Test

**Figure 8:**
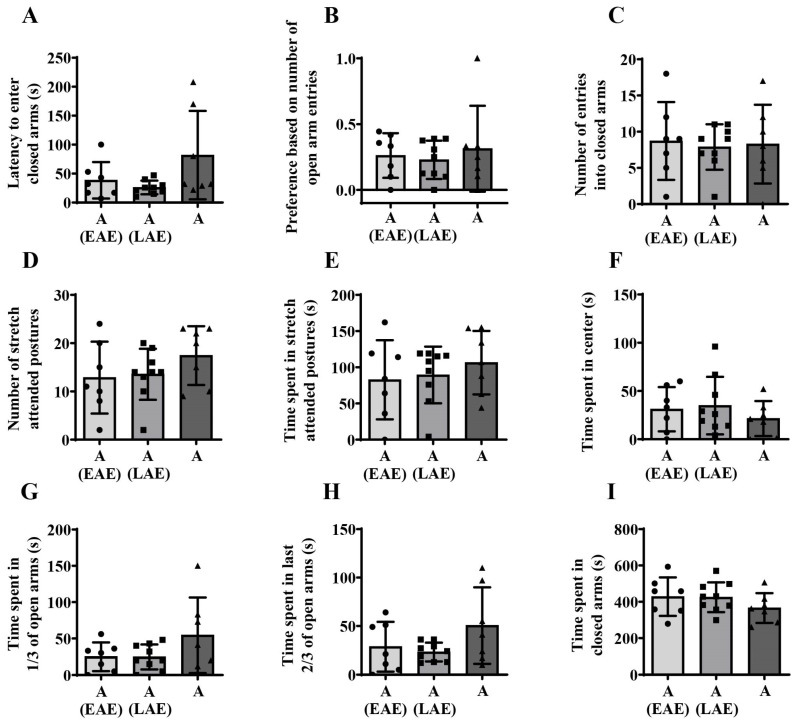
Influence of novelty-related **e**arly **a**dolescent **e**xperience (EAE) or **l**ate **a**dolescent **e**xperience (LAE) on adult (A) behavioral outcome in the elevated plus maze test. The data are represented as mean ± SD, with individual data plots along the column bars. Parameters analyzed were latency to enter closed area (**A**), preference based on number of entries in open arms (**B**), number of entries in closed arms (**C**), number of stretch-attended postures (**D**), time spent in stretch-attended postures (**E**), time spent in center (**F**), time spent in 1/3 of open arms (**G**), time spent in last 2/3 of the open arms (**H**), and time spent in the closed arms (**I**). No statistically significant differences were detected. Statistical comparisons and exact *p*-values are provided in Supplementary Table S2.

No significant influence of prior novelty-related adolescent experience with novel environments was observed in the latency to enter the closed arms of EPM (Figure 8A), preference based on the number of entries in open arms (Figure 8B), number of entries in closed arms (Figure 8C), number of stretch-attended postures (Figure 8D), time spent in stretch-attended postures (Figure 8E), time spent in center (Figure 8F), time spent in 1/3 of open arms (Figure 8G), time spent in last 2/3 of the open arms (Figure 8H), and time spent in the closed arms (Figure 8I).

## 4. Discussion

The obtained data suggest distinct manners of experiencing environmental novelty at different stages of adolescence and reveal how such experiences influence later behavioral/emotional outcomes. While late adolescent animals were much more sensitive to novelty exposure, displaying significant discomfort in aversive environments, early adolescent rats had a more moderate reaction, showing similarities in novelty-related responses to other maturational stages in a task- and parameter-specific manner. Overall, adolescent response to novelty was influenced by spatial, rather than nonspatial, aspects of the environment. Experience acquired in adolescence, particularly in late adolescence, influenced slight and stage-specific behavioral changes in adulthood.

It is widely accepted that adolescence represents a developmental stage during which animals need to embrace the risk and overcome the discomfort of leaving the nest and venture away in pursuit of new territories and opportunities, despite the dangers they must face on that path [47]. Consequently, it is anticipated that, in comparison to adults, adolescents will exhibit an elevated predisposition toward risk-taking behavior [48] and a preference for novelty [49,50], especially for spatial novelty [16]. However, studies have shown that when adolescent rats are exposed to novelty, they display varying anxiety-like responses—appearing more, less, or equally anxious compared to adult rats, mostly depending on the specific test employed [24,25,26]. By using multiple test methodology based on the same approach/avoidance paradigm [25,38], we intended to more precisely determine the behavioral response to novelty during maturation of male Wistar Han rats, knowing that strain and methodology differences may affect reproducibility of the results [51,52].

A decline in stereotypy-like behavior among adolescent rats in this study indicates that they experience less discomfort in this setting, given that such behavior is typically associated with lower agitation levels [53]. Nevertheless, reduced vertical activity (an important indicator of exploratory activity and a valuable marker of environmental novelty [54,55]) in this development stage shows that adolescents are less willing to investigate NA. Moreover, late adolescent animals show increased avoidance of the central area of NA. These results suggest that adolescent animals, compared to their adult counterparts, react more cautiously to the open areas (considered to be the surroundings with increased risk of predator attack [56,57]). Since adolescence is generally accepted to be development period characterized with increased novelty-seeking and risk-taking [48,49,50], this unexpected behavioral outcome can be interpreted in two ways: (1) adolescent animals are less motivated to investigate unsafe areas with no obvious rewarding potential (which is not to be misinterpreted as high levels of anxiety [58]); (2) a heightened defensive response to novel environments can be an outcome of increased anxiety, which is, in late adolescence, particularly manifested through thigmotaxis, the risk-averse strategy aimed to minimize exposure to potential threats in the unfamiliar environment [59]. Nevertheless, activity in the NA alone is not a sufficient parameter for assessing whether anxiety is the main drive behind observed behavioral outcomes, without additional measures obtained in other validated anxiety-related tests [43], especially because the NA is viewed as a less anxiety-provoking environment than the LDB or EPM [60,61,62].

In LDB and EPM, late adolescent animals exhibit higher avoidance of bright/lit and open/elevated surroundings and higher preference to enclosed and protected environments compared to other age groups, confirming an increase in anxiety-like behavior during this stage, which signifies an inverse U-shaped maturation profile of anxiety. Such a behavioral profile has not been typically observed in rodents [38], although it is common in the human population [63], which suggests that Wistar Han rats could serve as a valuable translational model for investigating the developmental trajectories of anxiety. Moreover, the lack of significant between-group differences in the number of entries in closed compartments (an indicator of general locomotor activity in EPM [64]) suggests that observed differences are not to be associated with overall hypoactivity in late adolescence, as might be assumed by behavioral changes in NA. Additionally, our findings indicate that adolescent rats display distinct behavioral patterns in risk assessment when confronted with unfamiliar environments. Both early and late adolescent groups exhibited a reduction in stretch-attended postures in the EPM, indicative of decreased risk-assessment behavior [65]. However, only early adolescent rats demonstrated an increase in time spent on the center, the area of the EPM associated with decision-making processes [66,67,68], which may represent a compensatory strategy for the diminished risk assessment reflected by fewer stretch-attended postures [69]. In line with that observation is that the developmental pattern of risk assessment observed in the LDB followed a U-shaped trajectory across age, showing the lowest expression of stretch-attended behavior during late adolescence, while levels were similar between early adolescents and adults. Overall, these findings indicate that early adolescent behavioral responses to novelty were not distinctly defined, displaying either similarities to late adolescent or adult patterns or exhibiting unique, stage-specific characteristics, depending on the parameters assessed and the environmental context of exposure. On the other hand, late adolescent responses to novelty are quite consistent across various spatial environments and characterized by increased anxiety and accompanied by lower locomotor, exploratory and risk-assessment activity, confirming an elevated trait anxiety profile in this developmental stage.

Novelty-related experiences gathered in adolescence influenced subtle yet noticeable behavioral changes in adulthood that slightly vary depending on the specific stage of adolescence during which the novelty-related experience occurred. In comparison to inexperienced adult animals, only adults exposed to the new environments in late adolescence displayed increased aversion to the center of NA. It is difficult to assume that this thigmotaxic behavior was due to explicit memory of past events since spatial memory tends to have a relatively limited duration in rodents, measured in days [70]. Moreover, a similar level of exploratory activity seen in both experienced and inexperienced adults implies that the experienced animals lack full awareness of their prior surroundings. It is more likely that the observed patterns of avoidance behavior can be attributed to the influence of an emotional memory trace [71], which remains after a robust emotional response to aversive environments in late adolescence. Such a form of implicit memory heavily relies on the normal functioning of the prefrontal cortex and amygdala, whose function and interconnectivity are particularly sensitive to exposure to stress during adolescence [72]. Late adolescence has been identified as a developmental stage particularly sensitive to exposure to stressful stimuli, which may influence heightened levels of anxiety later in adulthood [30,73]. However, changes in adult behavior in NA influenced by late adolescent novelty-related experiences may not be directly related to anxious response, as there were no discernible differences in anxiety-like behavior observed in the EPM and LDB tests between adults with and without prior experience. Even though a decrease in the latency to enter the dark compartment of LDB may suggest heightened anxiety, some authors recommend using latency as an additional measurement for anxiety only due to its unreliability [14]. As noted earlier, it is questionable whether exposure to an open arena is an adequate approach to measuring anxiety-like behavior in rodents [43]. Nevertheless, it should be noted that the early adolescent stage seems to be more flexible when adapting to low stress environmental novelty, leaving negligible carry-over effects into adulthood.

In contrast, reduced stereotypy-like behavior in experienced animals indicates that prior novelty-related experience may even, to some extent, relinquish agitation of being exposed to aversive environments in the future. Moreover, the number of stretch-attended postures, which is shown to be in high correlation with the circulating level of corticosterone [74] that is accountable for the appetitive properties of stress [75], is also reduced after prior exposure to novelty. Even though exposure to novel environments is regarded as a mild stressor [31], we showed that it can elicit varying degrees of behavioral response depending on adolescent stages when exposure occurred. Nonetheless, we also showed that such experience influences behavioral changes that can be associated with positive outcomes in adulthood. These findings extend on earlier research that suggests exposure to acute mild stressors may be even beneficial to the animals in captivity [33].

In the novel object exposure test, all age groups show a similar behavioral response, and prior novelty-related experience did not influence changes in novel object exploration in adulthood. Although the novel object exploration test is used to assess both novelty-seeking and anxiety-like behavior, it is questionable whether rats really express neophobia toward an object in a familiar environment [13]. Moreover, it seems that a novel object reduces the aversion of open space when presented in the center field [13]. Nevertheless, our study indicates that environmental/spatial novelty could be more influential for animal behavioral development than nonspatial novelty. For encoding and processing spatial information, normal activity of the hippocampus is of utmost significance [76,77]. Since the hippocampus undergoes significant alteration in adolescence, it is vulnerable to stressful stimuli, which may, in these developmental stages, induce changes in neural plasticity and neurogenesis and, in turn, influence atypical cognitive (spatial and nonspatial) [78] and emotional outcomes (anxiety and depression) [79]. Aberrant hippocampal activity has been identified as a crucial factor in defining animals with low novelty responsiveness, and is often characterized by elevated levels of anxiety and depression-like behavior [80]. However, acute mild stress is associated with an increase in hippocampal neurogenesis and neural plasticity and is accompanied by improved cognition, indicating that “a little stress can be a good thing” [33]. Therefore, more comprehensive research is required to examine how exposure to novelty during adolescence affects structural and functional alterations across various brain regions, particularly within the hippocampus, and how these alterations relate to the subsequent behavioral outcomes.

Overall, these findings point to the possibility of reusing experienced animals, particularly those exposed to novelty in early adolescence, in subsequent experimental procedures, thus supporting the increased imperative to reduce the number of animals used in studies [81,82]. This methodological approach enables the evaluation of individual emotional reactions to unpleasant spatial environments and allows for the identification of animals with low or high novelty responsiveness [80], as far as early adolescence, without causing undesirable behavioral outcomes in later maturational stages. However, it should be noted that novelty-related experience acquired in late adolescence may still influence behavior in less anxiogenic settings. In addition, short-term exposure to aversive environments in adolescence influences behavioral changes that may be associated with positive outcomes in adulthood, including reduced agitation. The observed decrease in agitation after short exposure to spatially aversive environments suggests that this approach could be a useful addition to traditional environmental enrichment strategies, which are usually object-focused and more nonspatial in nature [83]. However, further research is needed to better understand its long-term effects and potential applications in animal welfare protocols.

One limitation of this study is the relatively small sample size. Although group sizes were determined based on the relevant literature [35,36,37,38,39,40,41,42] and in accordance with the principle of reducing animal use, more extensive research across diverse species, strains, and larger cohorts is needed to confirm these findings and to allow more comprehensive general conclusions to be drawn. Another limitation of the study is the exclusion of female rats from the experimental procedure. Although some studies found no association between the estrus cycle and behavioral changes in rats, others recommend caution and point to the need to examine behavioral changes in all phases of the estrus cycle [84,85]. This was a challenge for our methodological approach, as the duration of some estrus phases in rats is measurable in hours [86]; thus, more extensive research is needed in order to extrapolate the obtained findings to females. Additionally, the order of behavioral tests in the battery was determined based on standard practice [25,38,39,40] and on evidence from the literature regarding potential confounding effects of prior test exposure [43,44]. Although the test order is theoretically based on a progression of increasing anxiety-provoking intensity, empirical studies providing direct behavioral and physiological validation of this assumption are scarce, underscoring the need for further investigation. Finally, forthcoming studies will investigate how adolescent exposure to novel environments induces structural and functional modifications across different brain regions, with an emphasis on the hippocampus and prefrontal cortex, and how these neural adaptations relate to subsequent behavioral outcomes.

## 5. Conclusions

In conclusion, findings in this study provide a clearer perspective on revealing developmental stage-specific differences in behavioral response to novelty in male Wistar Han rats. Maturational differences were most pronounced and influential in late adolescence. This study highlights the importance of experiencing spatial aspects of environmental novelty in adolescence, showing the nuanced influence of earlier novelty experiences on adult behavior—including its beneficial effects. It also indicates the feasibility of reusing previously tested adolescent animals in future experiments, supporting the ethical guidelines. Overall, the results obtained in this study represent novel insights into understanding how novelty-related experiences acquired in adolescence affect behavioral changes later in life. Regarding the lack of data on this topic, our results are of great importance, but more extensive research involving different species, strains, and larger cohorts of both sexes is needed.

## Data Availability

The original contributions presented in the study are included in the article/Supplementary Material; further inquiries can be directed to the corresponding author.

## Supplementary Materials

The following supporting information can be downloaded at https://www.mdpi.com/article/10.3390/ani15243552/s1, Table S1. Ontogeny of exploratory activity and anxiety-like behavior in novel surroundings; Table S2. Influence of adolescent experience with novel environments on exploratory activity and anxiety-like behavior in adulthood.

## Abbreviations

The following abbreviations are used in this manuscript:

EA	Early adolescence
LA	Late adolescence
A	Adult
NA	Novel arena (test)
NOE	Novel object exposure tests
LDB	Light/dark box (test)
EPM	Elevated plus maze (test)
NO	Novel object
EAE	Early adolescent experience
LAE	Late adolescent experience
PND	Postnatal day
BH	Benjamini–Hochberg correction
