# Supplementary material for: Experiencing Novelty in Adolescence and the Influence of Prior Novelty-Related Experiences on Adult Behavioral Outcomes in Wistar Han Rats
Source: Animals (Basel). 2025 Dec 10;15(24):3552. doi: 10.3390/ani15243552 (PMC12729729; doi:10.3390/ani15243552)
Supplement: Supplementary file 1 [file animals-15-03552-s001.zip › animals-3974269-supplementary.pdf]

Supplementary material.

Early adolescent group (EA; n=7), Late adolescent group (LA; n=9), Adult group (A; n=7)
The normality of data sets was estimated by the Shapiro-Wilk's test.
The accepted level of significance was $p \leq 0.05$ for all tests.
One-way ANOVA, followed by a post hoc unequal N HSD test (normal distribution).
Kruskal-Wallis H test, followed by the Mann–Whitney U-test for pairwise comparisons (no normal distribution).
Calculation of effect sizes and confidence intervals was not performed, as these estimates may be unreliable or misleading due to relatively small and unequal sample sizes for such type of analysis.

Table S1. Ontogeny of exploratory activity and anxiety-like behavior in novel surroundings

Figure		Statistical analysis		Benjamini-Hochberg (BH) correction	Post hoc test			
novel rectangular arena	**1A** Locomotor activity	One way ANOVA	$F(2,20) = 4.257$; $p = 0.029$	$p = 0.067$ adjusted	not applicable			
	**1B** Vertical activity	One way ANOVA	$F(2,20) = 30.226$; $p < 0.001$	$p = 0.007$ adjusted	Unequal N (HSD)	EA *vs.* A	LA *vs.* A	EA *vs.* MA
						$p < 0.001$	$p < 0.001$	$p = 0.717$
	**1C** Stereotypy-like behavior	One way ANOVA	$F(2,20) = 11.895$; $p < 0.001$	$p = 0.007$ adjusted	Unequal N (HSD)	EA *vs.* A	LA *vs.* A	EA *vs.* LA
						$p < 0.001$	$p = 0.007$	$p = 0.460$
	**1D** Time spent in center	Kruskal-Wallis ANOVA	$H (2, N= 23) =8.966$; $p =0.011$	$p = 0.035$ adjusted	Mann-Whtiney U test	EA *vs.* A	LA *vs.* A	EA *vs.* LA
						$p = 0.055$	$p = 0.004$	$p = 0.427$
novel object in a familiar arena	**2A** Latency to approach the NO	Kruskal-Wallis ANOVA	$H (2, N= 23) =3.393$; $p = 0.183$	$p = 0.260$ adjusted	not applicable			
	**2B** Number of approaches to NO	Kruskal-Wallis ANOVA	$H (2, N= 23) =2.505$; $p =0.286$	$p = 0.381$ adjusted	not applicable			
	**2C** Time spent in NO exploration	Kruskal-Wallis ANOVA	$H (2, N= 23) =2.317$; $p = 0.314$	$p = 0.406$ adjusted	not applicable			
light/dark box	3A Latency to enter dark compartment	Kruskal-Wallis ANOVA	$H (2, N= 23) =6.932$; $p = 0.031$	$p = 0.068$ adjusted	not applicable			

	3B<br>Number of stretch attended postures	One way ANOVA	F(2,20) = 9.285;<br>p = 0.001	p = 0.007 adjusted	Unequal N (HSD)	EA *vs.* A<br>p = 0.471	LA *vs.* A<br>p = 0.034	EA *vs.* LA<br>p = 0.003
	3C<br>Time spent in stretch attended postures	Kruskal-Wallis ANOVA	H (2, N= 23) = 10.433;<br>p = 0.005	p = 0.022 adjusted	Mann-Whtiney U test	EA *vs.* A<br>p = 0.949	LA *vs.* A<br>p = 0.001	EA *vs.* LA<br>p = 0.005
	3D<br>Number of entries in light compartment	Kruskal-Wallis ANOVA	H (2, N= 23) = 9.906;<br>p = 0.007	p = 0.026 adjusted	Mann-Whtiney U test	EA *vs.* A<br>p = 0.085	LA *vs.* A<br>p = 0.153	EA *vs.* LA<br>p = 0.017
	3E<br>Time spent in the light compartment	Kruskal-Wallis ANOVA	H (2, N= 23) = 9.892;<br>p = 0.007	p = 0.026 adjusted	Mann-Whtiney U test	EA *vs.*A<br>p = 0.141	LA *vs.* A<br>p = 0.039	EA *vs.* LA<br>p = 0.023
	3F<br>Time spent in dark compartment	Kruskal-Wallis ANOVA	H (2, N= 23) = 8.224;<br>p = 0.016	p = 0.047 adjusted	Mann-Whtiney U test	EA *vs.* A<br>p = 0.277	LA *vs.* A<br>p = 0.006	EA *vs.* LA<br>p = 0.039
elevated plus maze	**4A**<br>Latency to enter the closed arms	Kruskal-Wallis ANOVA	H (2, N= 23) =3.614;<br>p = 0.164	p = 0.241 adjusted	not applicable			
	**4B**<br>Preference based on number of entries in the open arms	Kruskal-Wallis anova	H (2, N= 23) =7.129;<br>p = 0.028	p = 0.067 adjusted	Mann-Whtiney U test	EA *vs.* A<br>p = 0.142	LA *vs.* A<br>p = 0.290	EA *vs.* LA<br>p = 0.013
	**4C**<br>Number of entries in the closed arms	Kruskal-Wallis anova	H (2, N= 23) =5.696;<br>p = 0.058	p = 0.121 adjusted	not applicable			
	**4D**<br>Number of stretch attended postures	One way anova	F(2,20) = 1.439;<br>p = 0.261	p = 0.359 adjusted	not applicable			
	**4E**<br>Time spent in stretch attended postures	One way anova	F(2,20) = 8.502;<br>p = 0.002	p = 0.011 adjusted	Unequal N (HSD)	EA *vs.* A<br>p = 0.006	LA *vs.* A<br>p = 0.006	EA *vs.* LA<br>p = 1.000
	**4F**<br>Time spent in center	One way anova	F(2,20) = 11.508;<br>p = 0.001	p = 0.007 adjusted	Unequal N (HSD)	EA *vs.* A<br>p = 0.008	LA *vs.* A<br>p = 0.568	EA *vs.* LA<br>p = 0.001
	**4G**<br>Time spent in first third of open arms	One way anova	F(2,20) = 2.842;<br>p = 0.081	p = 0.146 adjusted	not applicable			

	**4H** Time spent in last two thirds of open arms	One way anova	F(2,20) = 7.967; p = 0.003	p = 0.015 adjusted	Unequal N (HSD)	EA *vs.* A	LA *vs.* A	EA *vs.* LA
						p = 0.891	p = 0.021	p = 0.008
	**4I** Time spent in closed arms	One way anova	F(2,20) = 11.029; p = 0.001	p = 0.007 adjusted	Unequal N (HSD)	EA *vs.* A	LA *vs.* A	EA *vs.* LA
						p = 0.855	p = 0.007	p = 0.002

Table S2. Influence of adolescent experience with novel environments on exploratory activity and anxiety-like behavior in adulthood

Figure		Statistical analysis		Benjamini-Hochberg (BH) correction	Post hoc test			
novel rectangular arena	**5A** Locomotor activity	Kruskal-Wallis ANOVA	H (2, N= 23) =7.882; p = 0.019	p = 0.052 adjusted	not applicable			
novel rectangular arena	**5B** Vertical activity	One way ANOVA	F(2.20) = 2.819; p = 0.083	p = 0.146 adjusted	not applicable			
novel rectangular arena	**5C** Stereotypy-like behavior	One way ANOVA	F(2.20) = 8.681; p = 0.002	p = 0.011 adjusted	Mann-Whtiney U test	EAE *vs.*A	LAE *vs.* A	EAE *vs.* LAE
						p = 0.042	p = 0.003	p = 0.423
novel rectangular arena	**5D** Time spent in center	Kruskal-Wallis ANOVA	H (2, N= 23) =9.981; p =0.001	p = 0.007 adjusted	Mann-Whtiney U test	EAE *vs.*A	LAE *vs.* A	EAE *vs.* LAE
						p = 0.006	p = 0.020	p = 0.443
novel object in a familiar arena	**6A** Latency to approach the NO	Kruskal-Wallis ANOVA	H (2, N= 23) = 0.789; p = 0.674	p = 0.741 adjusted	not applicable			
novel object in a familiar arena	**6B** Number of approaches to NO	One way ANOVA	F(2.20) = 2.012; p = 0.160	p = 0.240 adjusted	not applicable			
novel object in a familiar arena	**6C** Time spent in NO exploration	One way ANOVA	F(2.20) = 3.036; p = 0.070	p = 0.140 adjusted	not applicable			

light/dark box	**7A** Latency to enter the dark compartment	Kruskal-Wallis ANOVA	H (2, N= 23) = 7.623; p = 0.022	p = 0.057 adjusted	not applicable			
	**7B** Number of stretch attended postures	One way ANOVA	F(2,20) = 6.295; p = 0.008	p = 0.027 adjusted	Unequal N (HSD)	EAE *vs.* A	LAE *vs.* A	EAE *vs.* LAE
						p = 0.038	p = 0.012	p = 0.851
	**7C** Time spent in stretch attended postures	Kruskal-Wallis ANOVA	H (2, N= 23) = 4.034; p = 0.133	p = 0.209 adjusted	not applicable			
	**7D** Number of entries in light compartment	Kruskal-Wallis ANOVA	H (2, N= 23) = 0.259; p = 0.879	p = 0.900 adjusted	not applicable			
	**7E** Time spent in the light compartment	Kruskal-Wallis ANOVA	H (2, N= 23) = 1.869; p = 0.393	p = 0.456 adjusted	not applicable			
	**7F** Time spent in dark compartment	Kruskal-Wallis ANOVA	H (2, N= 23) = 1.863; p = 0.394	p = 0.456 adjusted	not applicable			
elevated plus maze	**8A** Latency to enter the closed compartment	Kruskal-Wallis ANOVA	H (2, N= 23) = 4.243; p =0.112	p = 0.189 adjusted	not applicable			
	**8B** Preference based on number of entries in the open area	Kruskal-Wallis ANOVA	H (2, N= 23) =0.256; p =0.880	p = 0.900 adjusted	not applicable			
	**8C** Number of entries in the closed arms	One way ANOVA	F(2,20) = 0.063; p = 0.940	p = 0.940 adjusted	not applicable			
	**8D** Number of stretch attended postures	One way anova	F(2,20) = 1.116; p = 0.347	p = 0.425 adjusted	not applicable			
	**8E** Time spent in stretch attended postures	One way anova	H (2, N= 23) =0.638; p =0.727	p = 0.780 adjusted	not applicable			

	**8F** Time spent in center	One way anova	F(2,20) = 0.600; p = 0.559	p = 0.631 adjusted	not applicable
	**8G** Time spent in first third of open area	One way anova	F(2,20) = 2.842; p = 0.081	p = 0.146 adjusted	not applicable
	**8H** Time spent in last two thirds of open area	One way anova	F(2,20) = 2.263; p = 0.130	p = 0.209 adjusted	not applicable
	**8I** Time spent in closed area	One way anova	F(2,20) = 1.113; p = 0.348	p = 0.425 adjusted	not applicable
